# Renal transplant nephrolithiasis: Presentation, management and follow‐up with control comparisons

**DOI:** 10.1002/bco2.436

**Published:** 2024-09-10

**Authors:** Maxwell Sandberg, Adam Cohen, Megan Escott, Claudia Marie‐Costa, Davis Temple, Rainer Rodriguez, Alex Gordon, Anita Rong, Brian Andres‐Robusto, Emily H. Roebuck, Emily Ye, Gavin Underwood, Arjun Choudhary, Wyatt Whitman, Christopher J. Webb, Robert J. Stratta, Kyle Wood, Dean Assimos, Majid Mirzazadeh

**Affiliations:** ^1^ Department of Urology Atrium Health Wake Forest Baptist Medical Center Winston‐Salem North Carolina USA; ^2^ Wake Forest University School of Medicine Winston‐Salem North Carolina USA; ^3^ Edward Via College of Osteopathic Medicine Blacksburg Virginia USA; ^4^ Tufts University School of Medicine Boston Massachusetts USA; ^5^ Section of Transplantation, Department of Surgery Atrium Health Wake Forest Baptist Medical Center Winston‐Salem North Carolina USA; ^6^ Department of Urology University of Alabama Birmingham Medical Center Birmingham Alabama USA

**Keywords:** renal transplant, risk factor, stone, treatment, urology

## Abstract

**Objectives:**

To analyse the presentation, management and long‐term outcomes of renal transplant patients who formed kidney stones in their allograft. The secondary aim was to identify risk factors for stone formation in this cohort.

**Materials and Methods:**

Patient information from an institutional renal transplant database was used to identify individuals who both did and did not form kidney stones following renal transplantation. Computerized tomography (CT) imaging was used to make the diagnosis of kidney stones and measure stone size. Age‐ and gender‐matched controls never forming a stone in their allograft were used for comparative analysis to identify risk factors for stone formation in transplant patients.

**Results:**

A total of 8835 transplant patients were included in the study, of which 128 (1.4%) formed a kidney stone in their allograft after surgery. The mean time to kidney stone identification was 6.2 years, and the mean number of stones formed was 1.7, with a mean maximum size dimension on a CT scan of 5.7 mm per stone. A total of 26 patients were subjected to stone‐removing procedures, the most common being ureteroscopy (42.3%). The primary intervention failed in eight patients requiring a secondary intervention, and percutaneous nephrolithotomy (PCNL) had the lowest success rate (60%). A total of 164 controls were identified. In comparison to controls, stone formers had lower serum calcium (*p* = 0.008), lower estimated glomerular filtration rates (*p* = 0.019), higher lymphocyte counts (*p* = 0.021) and greater rate of urinary tract infection (*p* = 0.003). Graft failure rates were the same (*p* = 0.524), but time to graft failure was significantly longer in stone patients compared with controls (*p* = 0.008).

**Conclusions:**

The rate of stone formation is low in transplant patients. Success rates for stone treatment vary based on the surgery selected, with PCNL being the worst. Graft survival rates were equivocal, but survival time was better in stone patients. Our analysis calls for further investigation of this important topic.

## 1 INTRODUCTION

Nephrolithiasis in those subjected to renal transplantation is rare.^1^, ^2^, ^3^, ^4^ There is a paucity of information regarding this subject. The diagnosis is typically made 1–6 years after transplant.^5^, ^6^ Associated symptoms have not been well characterized. Information regarding stone composition, size, location, associated risk factors and treatment outcomes is also sparse. Furthermore, the impact of this condition on patient survival and graft function is not well established.

Various factors driving the development of kidney stones in this cohort have been proposed. Kim et al. and Harper et al. have reported hypocitraturia as a major risk factor.^7^, ^8^ Bolen et al. have noted that both hypocitraturia and hyperoxaluria are driving stone formation in this cohort.^9^ Hyperparathyroidism, hypercalciuria and recurrent urinary tract infection (UTI) have also been linked to stone formation in renal transplant patients.^7^

The purpose of this study was to analyse the presentation, management and long‐term outcomes of renal transplant patients who formed kidney stones in their allograft. The secondary aim was to identify risk factors for stone formation in this cohort.

## 2 MATERIALS AND METHODS

After obtaining Institutional Review Board approval (IRB00093774), patient information from an institutional renal transplant database was used to identify those individuals who developed as well as those who did not form kidney stones following renal transplantation. All subjects either had their kidney transplant at our institution or had this procedure done elsewhere, with the majority of follow‐up at our centre from 1984 to 2023. The electronic medical record was utilized to collect demographic data, stone variables and long‐term outcomes. Computerized tomography (CT) imaging was used to make the diagnosis of kidney stones and measure stone size, which was defined as the maximal diameter of the stone(s). Follow‐up CT scans were used to track stone size changes over time after initial diagnosis in those where observation was initially undertaken. Most asymptomatic patients underwent CT imaging for other reasons, such as evaluation of peri‐graft fluid collections or hydronephrosis detected by ultrasonography. Any patients who received a renal transplant graft containing a stone(s) were excluded, as were those who formed stones in their native kidneys after transplantation. ‘Other’ reasons for end‐stage renal disease (ESRD) included posterior urethral valves, trauma, recurrent UTI, obstructive uropathy, kidney tumours and hemolytic uremic syndrome.

Symptoms/signs associated with kidney stone(s) were classified as fever, costovertebral angle (CVA) tenderness, abdominal pain, groin pain, dysuria, microscopic hematuria and gross hematuria. Microscopic hematuria was defined as >3 red blood cells/high‐powered field on urinalysis. Stone compositions determined by infrared spectroscopy and X‐ray diffraction were defined as the major component. Stone treatments labelled as ‘other’ included any form of open stone surgery or robotic stone surgery. The comparison group was those who did not have any International Classification of Diseases codes for calculus of the kidney, including those who had no CT scan evidence of nephrolithiasis in their transplant grafts. Age and gender matching, based on the size of the stone cohort prior to exclusion criteria being applied, was undertaken. Serum basic metabolic panel (BMP) and complete blood count (CBC) results were analysed with utilization of values closest to the date of stone identification. For the non‐stone‐forming group, BMP and CBC results closest to the mean time of stone identification after the transplant procedure were used. Graft donor patient information was not included in analysis as the majority of these patients did not have demographic and health information available in the electronic medical record for review. Independent samples *t*‐test was used to compare scaled variables, and Pearson's chi‐squared test was used for categorical variables between stone formers and non‐stone formers. Kaplan–Meier survival analysis with log‐rank testing was utilized to compare patient and graft survival rates between stone formers and non‐stone formers. Of note, two patients from the control group were excluded for survival analysis on graft failure because of a lack of consistent documentation in the medical record for the date of graft failure. All statistical analyses were conducted using SPSS Version 28 (Armonk, NY).

## 3 RESULTS

A total of 8835 transplant patients who met inclusion criteria were included in the study. Twenty‐eight patients were excluded secondary to receiving a renal transplant with a stone already in the graft and seven patients because of new stone formation in the native kidneys. One hundred and twenty‐eight individuals formed at least one stone in their graft after transplantation, with a prevalence of 1.4% (Table 1). Sixty‐seven were men and 61 were women. The mean time to kidney stone identification was 6.2 years following renal transplant. The mean number of stones formed was 1.7, with a mean maximum size dimension on CT scan of 5.7 mm per stone. Twenty (15.6%) of patients formed additional stone(s) after their primary diagnosis of kidney stones. Diabetes mellitus (DM; *N* = 40; 31.3%) was the primary cause of ESRD in stone‐forming patients, followed by hypertension (*N* = 19; 14.8%) and autosomal dominant polycystic kidney disease (ADPKD; *N* = 14, 10.9%)/focal segmental glomerular sclerosis (FSGS; *N* = 14, 10.9%). One hundred and fourteen patients underwent deceased donor transplantation, and 14 received a living donor kidney transplant. Fifty‐seven (44.5%) patients had a history of at least one UTI after renal transplantation. One hundred and nine of the stones were located in the renal collecting system and 19 in the ureter. Twenty‐nine (22.7%) patients experienced signs or symptoms from their stones, the most common being microhematruia in 16 (55.2%) of these patients.

**TABLE 1:** Kidney stone variables with control comparison. The table lists demographic, stone, management and follow‐up data on all patients who formed a kidney stone in the study. When applicable, control patient data are also listed with comparison using either independent samples *t*‐tests or chi‐squared tests and associated *p*‐values. Standard deviations are in parentheses for continuous variables and percentage of the cohort for categorical variables.

Variable	Stone former	Control	*p*‐value
*N*	128	164	‐
BMI (SD)	28.4 (6.4)	29.3 (6.5)	0.215
Age at transplant (years) (SD)	49.1 (15.5)	50.6 (14.2)	0.424
Female (%)	61 (48)	76 (46.3)	0.823
Race			
White (%)	73 (57)	90 (54.8)	0.423
Black (%)	48 (37.5)	61 (37.2)	
Latino (%)	2 (1.6)	5 (3)	
Asian (%)	1 (0.78)	1 (0.6)	
Alaskan Native (%)	1 (0.78)	7 (4.3)	
Other	3 (2.3)	0	
Diabetes (%)	61 (47.7)	88 (53.6)	0.288
Haemoglobin A1c (SD)	6.9 (1.3)	7.1 (1.5)	0.172
Active smoker (%)	54 (42.2)	66 (40.2)	0.873
Coronary artery disease (%)	29 (22.7)	33 (20.1)	0.247
Hyperlipidemia (%)	62 (48.4)	89 (54.2)	0.247
Cause of ESRD			
DM	40 (31.3)	56 (34.1)	‐
Hypertension	19 (14.8)	26 (20.3)	
ADPKD	14 (10.9)	15 (11.7)	
FSGS	14 (10.9)	12 (9.4)	
IgA nephropathy	6 (4.7)	9 (7)	
Chronic glomerulonephritis	7 (5.5)	11 (8.6)	
Lupus	4 (3.1)	3 (1.8)	
Membranous nephropathy	2 (1.6)	5 (3)	
Reflux	4 (3.1)	1 (0.8)	
Chemical exposure	1 (0.8)	2 (1.6)	
Scleroderma	2 (1.6)	0	
Other genetic causes	3 (2.3)	5 (3)	
Other causes	10 (7.8)	14 (8.5)	
Unknown	4 (3.1)	5 (3)	
Transplant type			
Deceased donor (%)	114 (89)	129 (78.6)	0.267
Living donor (%)	14 (11)	35 (21.4)	
Multiple transplants (%)	14 (10.9)	14/159 (8.8)	0.530
UTI after transplant	57 (44.5)	46 (28)	0.003
Stones found on first CT scan	1.7 (1.2)	‐	‐
Time from transplant to first stone formation (years)	6.2 (7.1)	‐	‐
Location of stone on CT scan			
Kidney (%)	109 (85)	‐	‐
Ureter (%)	19 (15)		
Mean maximal size of first stone (mm) (SD)	5.7 (4.2)	‐	‐
Stone increased in size over time (%)	25 (19.5)	‐	‐
Formed new stones after primary diagnosis (%)	20 (15.6)	‐	‐
Signs/symptomatic stone (%)	29 (22.7)	‐	‐
Fever (%)	4/29 (13.8)	‐	‐
CVA tenderness (%)	5/29 (17.2)	‐	‐
Groin pain ((%)	2/29 (6.9)	‐	‐
Nausea (%)	7/29 (24.1)	‐	‐
Abdominal pain (%)	7/29 (24.1)	‐	‐
Gross hematuria (%)	7/29 (24.1)	‐	‐
Microhematuria (%)	16/29 (55.2)	‐	‐
Dysuria (%)	10/29 (34.5)	‐	‐
Received treatment for stone (%)	26 (20.3)	‐	‐
Type of treatment			
SWL (%)	6 (23.1)	‐	‐
Ureteroscopy (%)	11 (42.3)		
PCNL (%)	5 (19.2)		
Other (%)	4 (15.4)		
Primary success rate			
SWL (%)	5 (83.3)	‐	‐
Ureteroscopy (%)	7 (63.6)		
PCNL (%)	3 (60)		
Other (%)	3 (75)		
Retreatment			
SWL (%)	0	‐	‐
Ureteroscopy (%)	1 (12.5)		
PCNL (%)	5 (62.5)		
Other (%)	2 (25)		
Stone composition			
Calcium oxalate (%)	6 (40)	‐	‐
Calcium phosphate (%)	1 (6.7)		
Struvite (%)	3 (20)		
Uric acid (%)	2 (13.3)		
Other (%)	3 (20)		
Graft failure	20 (15.6)	32 (19.5)	0.524
Time to graft failure (years)	9 (6.9)	4.8 (4)	0.008
Died (%)	32 (25)	40 (24.3)	0.905
Time to death from stone detection (years) (SD)	1.8 (2)	‐	‐
Time to death from transplant (years) (SD)	10.1 (7.4)	7.4 (7.6)	0.141

Abbreviations: ADPKD, autosomal dominant polycystic kidney disease; BMI, body mass index; CT, computerized tomography; CVA, costovertebral angle; DM, diabetes mellitus; ESRD, end‐stage renal disease; FSGS, focal segmental glomerular sclerosis; PCNL, percutaneous nephrolithotomy; SWL, shockwave lithotripsy; UTI, urinary tract infection.

Twenty‐six (20.3%) patients were subjected to stone‐removing procedures. The primary intervention failed in eight patients requiring a secondary intervention. The most common primary treatment was ureteroscopy in 11 (42.3%) patients, followed by shockwave lithotripsy (SWL) in 6 (23.1%), percutaneous nephrolithotomy (PCNL) in 5 (19.2%) and other stone surgery in 4 (15.4%) patients. The success rate of ureteroscopy was 63.6%, for SWL it was 83.3%, for PCNL it was 60% and 75% for other stone surgery. Of the eight failed treatments, 4 were ureteroscopy (50%), 2 were PCNL (25%), 1 was SWL (12.5%) and 1 was categorized as other stone surgery (12.5%). Moreover, on secondary intervention, 1 patient underwent ureteroscopy (12.5%), 0 SWL, 5 PCNL (62.5%) and 2 other stone surgeries (25%). One patient again failed their secondary intervention, and this was a patient who received other stone surgery. Stone composition results were available in 15 patients. Calcium oxalate was the most common predominant stone composition, present in six (40%) patients. Twenty (15.6%) patients experienced graft failure at an average of 9 years after transplant. Thirty‐two (25%) stone‐forming patients died during the study window at an average of 1.8 years after stone identification and 10.1 years from their transplant operation.

**FIGURE 1:**
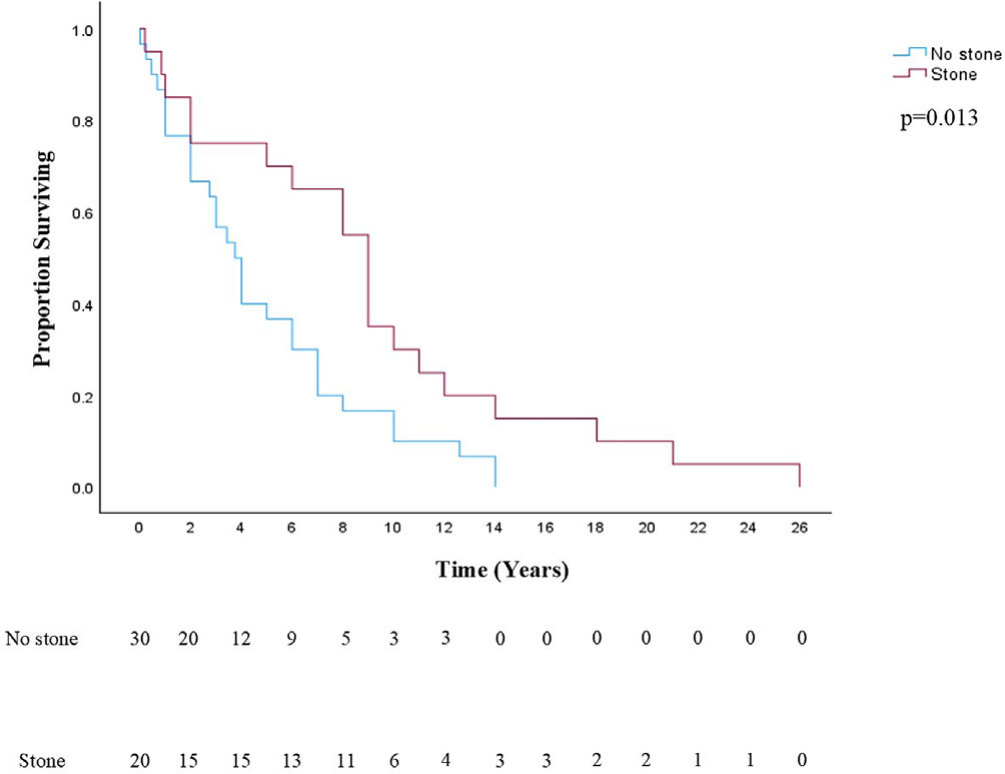
Graft survival after transplant. Kaplan–Meier survival curve comparing graft survival after transplantation in the stone‐forming (red) and control populations (blue). Using the log‐rank test, a significant difference was identified between groups favouring survival in the stone‐forming cohort (*p* = 0.013). Number at risk table is also provided below the survival curve.

**FIGURE 2:**
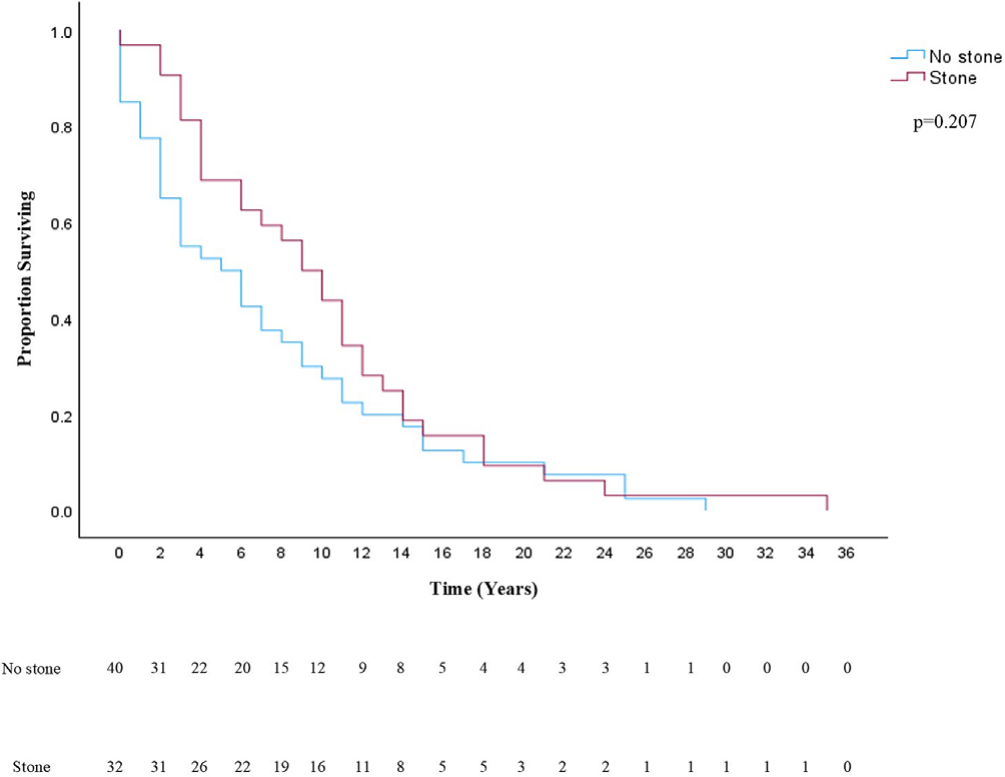
Patient survival after transplant. Kaplan–Meier survival curve comparing patient survival after transplantation in the stone‐forming (red) and control populations (blue). Using the log‐rank test, no significant difference was identified between groups (*p* = 0.207). Number at risk table is also provided below the survival curve.

One hundred and sixty‐four age‐ and gender‐matched non‐stone formers were used for comparative analysis. Of these, 129 (78.7%) underwent a deceased donor and 35 (21.3%) a living donor kidney transplant (*p* = 0.267; compared with study group). DM was the leading cause of ESRD in control patients (*N* = 56; 34.1%), followed by hypertension (*N* = 26; 15.9%) and ADPKD (*N* = 15; 9.1%). All patients had multiple follow‐up ultrasounds of their transplant graft, which did not show the development of kidney stones. Ninety‐seven (59.1%) patients also had a CT scan confirming the absence of stones after renal transplant. Baseline characteristics including body mass index (BMI; *p* = 0.215), age at transplant (*p* = 0.424), gender (*p* = 0.823), race (*p* = 0.423) and comorbidities including DM, hyperlipidemia and coronary artery disease (*p* > 0.05) were similar. Rate of UTI was significantly lower in controls (*N* = 46, 28%) compared with stone formers after transplant surgery (*p* = 0.003). Stone formers had lower serum calcium (*p* = 0.008), lower estimated glomerular filtration rates (GFRs; *p* = 0.019) and higher lymphocyte counts (*p* = 0.021) (Table 2). The rate of graft failure did not differ between stone formers and controls (*p* = 0.524). Time to graft failure was significantly longer in stone patients, at a mean of 9 years (Figure 1; *p* = 0.013). Forty (24.4%) non‐stone‐forming patients died after transplant as compared with 32 without stones (25%, *p* = 0.905). Patient survival after transplant was also similar between the groups (Figure 2; *p* = 0.207).

**TABLE 2:** Laboratory value comparisons. The following table compares complete blood count (CBC), basic metabolic panel (BMP) and urine labs taken as close to the time of stone identification in stone forming patients and labs taken at the mean time to stone formation after transplant in controls. Means are listed with standard deviations in parentheses. Comparisons were done using independent samples *t*‐test with associated *p*‐value.

Laboratory value	Stone former	Control	*p*‐value
Phosphorous (SD)	3.5 (1.3)	4.2 (7.2)	0.283
Magnesium (SD)	1.8 (0.5)	1.8 (0.3)	0.811
Vitamin D (SD)	34 (18.3)	30 (14.8)	0.282
Parathyroid hormone (SD)	276.5 (277.9)	232.1 (330.8)	0.424
Creatinine (SD)	2.8 (2.8)	2.4 (2.7)	0.255
Calcium (SD)	9.1 (0.8)	9.3 (0.7)	0.008
GFR (SD)	38.1 (19.6)	43.6 (19.4)	0.019
Urine protein (SD) (mg/dL)	128.4 (358.4)	65.1 (133.7)	0.157
Urine creatinine (SD)	110.7 (62.8)	112 (60.1)	0.880
Platelets (SD) (10^3^)	200.3 (73.5)	204.6 (90.5)	0.664
Lymphocyte (SD) (10^3^)	8.5 (7.5)	1.5 (2.9)	0.021
Neutrophil (SD) (10^3^)	5.4 (3.1)	6.4 (9.8)	0.229
Monocyte (SD) (10^3^)	0.6 (0.3)	0.8 (1.5)	0.092

Abbreviation: GFR, glomerular filtration rate.

## 4 DISCUSSION

We present a large series of renal transplant patients who went on to develop kidney stones postoperatively, with data on presentation, management and long‐term follow‐up. In comparison to prior reports, our cohort's mean time to stone formation was approximately 6 years, at the upper end of reported ranges of 1–6 years.^5^, ^6^, ^10^ There was also a higher proportion of female transplant patients forming stones in comparison to those reported for the general population; a finding reported by others.^2^, ^10^, ^11^, ^12^, ^13^ A minority of our patients were subjected to multiple renal transplants, and some formed a stone in their subsequent transplant graft as opposed to their initial transplant graft. Of the entire stone cohort, 22.7% were symptomatic and/or had clinical signs of kidney stones, the most common sign being microscopic hematuria. In other reports, upwards of 45% of transplant patients experienced symptoms from their stones.^14^ Most allograft kidney stones in this study were discovered incidentally when the patient was undergoing a CT scan for another reason.

A mean of 1.7 stones were seen on the first CT, with a mean maximal stone size of 5.7 mm. This is a smaller average stone size than what has been noted in other publications.^7^, ^15^, ^16^ Most stones occurred in the donor kidney graft and a minority in the transplant ureter; again consistent with prior reports.^13^ Of all patients in the stone cohort, 20.3% were subjected to stone‐removing interventions. The most common treatment modality used was ureteroscopy, which was performed in a retrograde fashion in all cases. This is also the most common procedure previously utilized in this cohort, with some utilizing an antegrade approach.^10^, ^17^ SWL was the second most common procedure, followed by PCNL. Eight (30.7%) patients failed their primary treatment, of which ureteroscopy was the most frequently failed primary intervention. Interestingly, PCNL was mostly used as a salvage treatment, which has previously been reported to be more effective than ureteroscopy and SWL.^10^, ^17^ A total of 15.6% of patients experienced graft failure during the study window, on average 9 years post‐transplant, consistent with accepted rates in the general transplant population.^18^

A secondary aim of this study was to identify risk factors for stone formation in the transplant population. Basic demographic characteristics such as age, gender, race, BMI and comorbidities were equivalent between the two groups. Laboratory data were also studied, and significant differences between stone formers and controls were seen. This included a lower serum calcium, a lower estimated GFR and a higher absolute lymphocyte count compared with controls. Prior risk factors for transplant stones have been shown to be hyperparathyroidism, hypercalcemia, hypercalciuria, hyperoxaluria, hypocitraturia and recurrent UTI.^7^, ^16^ Although our finding of a lower calcium level in stone formers conflicts with previous literature, these studies do not include comparisons to an appropriately matched cohort, which may account for this difference. The greater lymphocyte count seen in our stone‐forming cohort is a novel finding. This could be related to the role that the immune system plays in kidney stone formation.^19^ Nevertheless, we recognize our laboratory findings of differences in calcium and lymphocyte counts between stone formers and non‐stone formers could be because of chance alone, and comparison of values at multiple time points would have been superior in drawing definitive conclusions. Interestingly, there was a greater occurrence of UTIs after transplantation in the stone group, although both groups had a fairly high UTI rate overall. UTI is well‐known as the most common infection after renal transplant, but a greater incidence in the stone group is worth highlighting as UTI is also an established risk factor for stone formation in the general population.^20^, ^21^ We also sought to compare patient and graft survival rates with controls. Patient survival rates were equivalent across both groups, but graft survival time was greater in stone formers. Others have examined graft failure after stone formation and found that the development of stones had no effect on failure rates.^3^, ^7^ Our study agrees with this finding but goes a step further as we found evidence that it may be protective from graft failure. We hypothesize that the stone(s) themselves are not protecting the graft, but rather more frequent and diligent follow‐up may account for this outcome. Alternatively, given that the mean time to stone detection was 6.2 years, the apparent graft survival benefit may just be the result of selection bias in the stone‐forming group towards grafts that functioned longer compared with the non‐stone‐forming cohort or subtle other differences between groups.

We recognize the inherent limitations of a retrospective review with relatively small numbers in the study cohorts and reliance on the information in the electronic medical record being correct. Not all of the non‐stone‐forming groups were subjected to CT, and some of those patients could have been harbouring stones. However, all the non‐stone‐forming group were also subjected to multiple post‐transplant graft ultrasonographies, which would likely have prompted CT imaging if stones were suspected. Further, we were unable to appropriately comment on patients who may have passed their stone(s) spontaneously, as this information was not readily available in the electronic medical record. We also did not account for the effect of patient diet, environment and genetics, all of which impact stone formation and were not readily available in the medical record. Inclusion of 24‐h urinary kidney stone risk data would have strengthened this study. However, only three stone‐forming patients underwent such studies, and thus this was not utilized to define stone risk. A future effort to undertake such urinary studies will be made. This may help identify those who are at risk for stone recurrence and may allow for the implementation of dietary and medical strategies for stone prevention in this cohort. In addition, future studies should be undertaken to determine the stone recurrence rate in this cohort.

## 5 CONCLUSION

This single‐centre review of nephrolithiasis in renal transplant patients is one of the largest ever reported. We include data on patient characteristics, stone formation, management and follow‐up. Many of our findings are in line with prior literature, although our cohort had smaller stones and were less frequently symptomatic than other studies. Further, we compared stone‐forming patients to an appropriately matched non‐stone‐forming cohort to identify potential risk factors for kidney stone formation after renal transplantation. These findings add to a small body of literature and are applicable for a variety of physicians and practitioners treating kidney transplant patients. Our analysis calls for further investigation of this important topic.

## AUTHOR CONTRIBUTIONS

Maxwell Sandberg organized the project, helped in data collection and wrote the first draft of the manuscript. Adam Cohen helped in project organization and data collection and assisted in writing the first draft of the manuscript. Davis Temple, Claudia Marie‐Costa, Rainer Rodriguez, Alex Gordon, Anita Rong, Brian Andres‐Robusto, Emily Ye, Gavin Underwood, Arjun Choudhary, Adam Cohen and Emily H. Roebuck helped in organizing and obtaining data collection for the project. Wyatt Whitman helped in project conceptualization and data collection. Dean Assimos, Kyle Wood, Christopher J. Webb, Robert J. Stratta and Majid Mirzazadeh were senior organizing authors and assisted in manuscript drafting.

## CONFLICT OF INTEREST STATEMENT

The authors declare no conflicts of interest.
